# A Critical Review of the Impact of *SMARCA4* Mutations on Survival Outcomes in Non-Small Cell Lung Cancer

**DOI:** 10.3390/jpm14070684

**Published:** 2024-06-26

**Authors:** Peter Manolakos, Luigi Boccuto, Diana S. Ivankovic

**Affiliations:** Healthcare Genetics and Genomics PhD Program, Clemson University, Clemson, SC 29634, USA; lboccut@clemson.edu (L.B.); divanko@clemson.edu (D.S.I.)

**Keywords:** *SMARCA4*, mutation, *KRAS*, co-mutation, *KRAS*/*SMARCA4*, NSCLC, non-small cell lung cancer, survival, treatment outcome, immunotherapy

## Abstract

This critical review investigates the impact of SWI/SNF-related, matrix-associated, actin-dependent regulator of chromatin, subfamily A, member 4 (*SMARCA4*) mutations on survival outcomes in non-small cell lung cancer (NSCLC) through an analysis of 21 peer-reviewed articles. Survival analyses across this review demonstrated consistently worse outcomes for *SMARCA4*-mutated vs. *SMARCA4* wild-type NSCLC patients, specifically emphasizing class 1 truncating mutations as an independent factor for poor overall survival. In addition, this review explores the clinicopathologic characteristics of *SMARCA4* mutations and their impact on various treatment modalities, including immune checkpoint inhibitors (ICIs) both with and without Kirsten rat sarcoma viral oncogene homolog (*KRAS*) co-mutations. The potential ineffectiveness of ICI treatment in NSCLC is explored through the impact of *SMARCA4*/*KRAS* co-mutations on the tumor microenvironment. Moreover, this NSCLC review consistently reported statistically worse overall survival outcomes for *SMARCA4*/*KRAS* co-mutations than *SMARCA4* wild-type/*KRAS*-mutated cohorts, extending across ICIs, chemo-immunotherapy (CIT), and *KRAS* G12C inhibitors. Designing prospective clinical *SMARCA4*-mutated or *SMARCA4*/*KRAS* co-mutated NSCLC trials to evaluate targeted therapies and immunotherapy may lead to a better understanding of how to improve cancer patients’ outcomes and survival rates.

## 1. Introduction

Over the past twenty years, the Food and Drug Administration (FDA) has approved a minimum of 20 new molecular entities that target oncogenic driver mutations in non-small cell lung cancer (NSCLC) [1]. These targeted therapies have led to some NSCLC studies reporting a median overall survival (OS) over three years in the metastatic setting [2]. While more than fifty percent of non-squamous NSCLC patients may have a mutation with an FDA-approved therapy, there remains a distinct unmet medical need for those with epigenetic dysregulation that may be more challenging to target [1,3]. This need is particularly vital given that the five-year survival rate for Stage IV NSCLC patients in 2020 was only 8.2% [4].

This critical review analyzes the current literature on the impact of mutations in the SWI/SNF-related, matrix-associated, actin-dependent regulator of chromatin, subfamily A, member 4 (*SMARCA4*) gene, focusing on the survival outcomes for patients with NSCLC. Typically, patients with *SMARCA4*-deficient NSCLC clinically present with adenocarcinoma, larger invasive tumor size, a smoking history, and fewer epidermal growth factor receptor (EGFR) mutations (*p* < 0.05) [5,6]. In addition, *SMARCA4*-mutated NSCLCs are associated with a higher likelihood of negative or low programmed cell death ligand-1 (PD-L1) expression (*p* =0.03) and higher tumor mutational burden (TMB) as compared to wild-type (WT) cases (*p* < 0.001) [7,8].

*SMARCA4* mutations occur in approximately 5–7% of all human cancers and 7–11% of NSCLC cases [6,9,10,11]. Two classes of *SMARCA4* alterations are linked to epigenetic dysregulation [12]. Class 1 mutations encompass truncating mutations, fusions, and homozygous deletions, typically associated with protein loss and loss of function. Class 2 mutations involve missense mutations, which are suggested to employ dominant-negative or gain-of-function effects. Some reports indicate a loss of function leading to reduced accessibility and diminished chromatin remodeling activity, mainly observed in lung cancer [11]. In a comprehensive study of 4813 tumors from NSCLC patients, Schoenfeld et al. discovered that 212 patients (4% of the total population) exhibited class 1 *SMARCA4* alterations (52% of the *SMARCA4* variants) [8]. In contrast, 195 patients (4% of the total population) had tumors with class 2 *SMARCA4* alterations (48% of the *SMARCA4* variants) [8]. Of particular interest, *SMARCA4* mutations are associated with altered MYC gene expression, which is essential in regulating cell growth and proliferation [13].

*SMARCA4* is a tumor suppressor gene located on chromosome 19p13.2 that encodes the Brahma-Related Gene 1 (BRG1) protein, which is one of two mutually exclusive critical DNA-dependent ATPases (Brahma is the other) that regulates gene expression by altering the chromatin structure [14]. In addition, the BRG1 protein contains a bromodomain that plays a critical role in gene transcription and is essential in recognizing acetylated lysine residues on N-terminal tails of histones [14].

BRG1 is part of the mammalian-type switch/sucrose non-fermenting (mSWI/SNF) chromatin-regulatory complex (CRC) [15]. The mSWI/SNF remodeling complex is essential in regulating chromatin structures and contains approximately 10–12 subunits [16]. Utilizing energy obtained through ATP hydrolysis, the catalytic subunits of human mSWI/SNF change nucleosome positioning. This alteration modulates the accessibility of transcriptional machinery to DNA, ultimately impacting the activation or repression of specific genes. The mSWI/SNF CRC protein subunits of interest are AT-Rich Interaction Domain 1A (ARID1A), SWI/SNF Related, Matrix-Associated, Actin-Dependent Regulator of Chromatin, Subfamily B, Member 1 (SMARCB1), and BRG1. They are among the most studied CRCs and have higher rates of mutations in human cancers than other CRC subunits [17].

The most frequent co-mutations found in the Massachusetts General Hospital analysis of 29 *SMARCA4* patients that lacked BRG1 protein expression affected *TP53* (N = 17, 59%), *STK11* (N = 15, 52%), *KEAP1* (N = 12, 41%), and *KRAS* (N = 10, 34%) [6]. These co-mutation frequencies were validated in a more extensive Foundation Medicine data set with *SMARCA4* co-mutations in *TP53* (74%), *CDKN2A* (38%), *STK11* (34%), *KRAS* (26%), and *KEAP1* (15%) [6].

Further research is needed to unravel the intricate interplay between *SMARCA4*, chromatin remodeling, and tumorigenesis in non-small cell lung cancer. A comprehensive understanding of the nuanced interplay between *SMARCA4* and *KRAS* co-occurring mutations in the context of NSCLC is paramount, as *KRAS* mutations commonly co-occur with various mutations and have been found to have a poor prognosis across several NSCLC studies [18]. This review aims to summarize the clinical studies that have elucidated the potential implications of *SMARCA4* mutations and *SMARCA4*/*KRAS* co-mutations on survival outcomes when treating metastatic NSCLC patients across various treatments (i.e., chemotherapy and immune checkpoint inhibitors).

## 2. Materials and Methods

PubMed and the Web of Science were searched using the following keywords: “SMARCA4” and “NSCLC”. Boolean operators were used to connect specific search keywords. Peer-reviewed articles published in English between 2018 and 2023 were included, and duplicate records were removed. Articles were included if they contained any of the following concerning NSCLC patients with *SMARCA4* mutations vs. *SMARCA4* WT: OS hazard ratio (HR), progression-free survival (PFS) HR, median OS, or median PFS. Articles were excluded if they were solely pre-clinical or diagnostic. Articles were also excluded if the majority of *SMARCA4* lung cancer patients were diagnosed with non-NSCLC (i.e., small-cell), an undifferentiated tumor, or sarcomatoid *SMARCA4* NSCLC. Articles were loaded into Raayan [19] and reviewed by all authors. Discrepancies were discussed until a consensus was reached on included vs. excluded articles.

**Figure 1:**
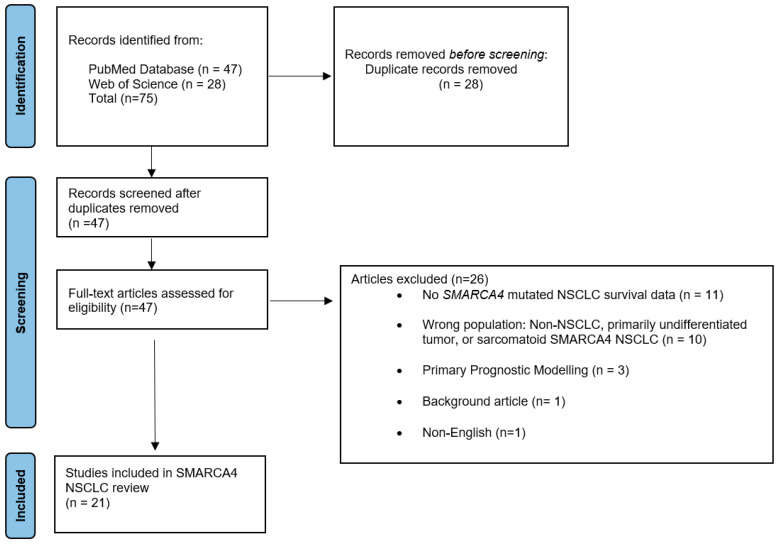
PRISMA flow diagram illustrating the study selection process.

The search strategy was as follows: (smarca4 [Title/Abstract]) AND (nsclc [Title/Abstract]) Filters: Full text, Humans, from 2018–2023. The study selection process is outlined in the PRISMA flow diagram (Figure 1) [20]. 

## 3. Results

### 3.1. Results and Description of Studies

In this critical review, 75 articles were identified by applying the search strategy. Twenty-eight articles were excluded and removed as duplicate records, and 47 articles were fully reviewed. Twenty-six articles were excluded due to the lack of *SMARCA4*-mutation NSCLC survival outcomes, primary prognostic modeling, lack of incorrect patient population (i.e., sarcomatoid histology), review articles, and being written in non-English.

Twenty-one studies were included and reviewed in this critical review of *SMARCA4* NSCLC. The included articles focus on survival outcomes across various patient categories, clinicopathologic characteristics, and immune checkpoint inhibitors (ICIs) cancer treatment. Moreover, a separate subheading focusing on *SMARCA4* and *KRAS* co-mutation survival outcomes in patients with NSCLC is included, as this has recently become a medically relevant topic in the lung cancer community. Table 1 summarizes the NSCLC mutation analyzed, treatment, outcome, and relevance of each study in this review.

### 3.2. SMARCA4 Mutations

Schoenfeld et al. examined the clinicopathologic characteristics in a multivariate NSCLC analysis and determined that both *SMARCA4*-mutated class 1 (N = 149) and class 2 (N = 143) had worse OS vs. WT (*p* < 0.001) using Kaplan–Meir methods [8]. In addition, class 1 *SMARCA4*-mutated patients had the worst OS compared to class 2 mutated or WT patients (N = 996) via a log-rank test analysis [8]. Furthermore, an ICI survival treatment analysis (N = 87) showed an OS improvement for *SMARCA4*-mutated patients receiving ICI treatment vs. without ICI treatment regardless of class 1 or class 2 mutation status (*p* = 0.01). However, there was no significant difference in PFS (*p* = 0.74) or OS (*p* = 0.35) for *SMARCA4*-mutated patients receiving ICIs when comparing class 1 vs. class 2 alterations in an unadjusted analysis [8].

Both studies from Alessi et al. further support *SMARCA4* mutations as an independent factor that leads to worse outcomes for NSCLC patients [21,22]. In one of the most extensive NSCLC studies of patients with *SMARCA4* mutations who received chemo-immunotherapy (CIT), Alessi et al. reported that patients with non-squamous histology who had *SMARCA4* mutations (N = 114) vs. WT (N = 593) had significantly worse survival: PFS (2.7 versus 6.1 months, HR: 1.62; *p* < 0.001), and OS (8.1 versus 15.0 months, HR: 1.70; *p* < 0.001) from the start of CIT [22]. A multivariable analysis using inverse probability weighting was conducted to address potential selection bias due to the absence of Programmed Death Ligand-1 (PDL-1) reporting. The analysis revealed that confirmed *SMARCA4* mutations independently correlated with reduced survival: PFS (*SMARCA4*-mutated HR: 1.61; *p* < 0.001), OS (*SMARCA4*-mutated HR: 1.66; *p* <0.001) to CIT [22].

A separate Alessi et al. study reported better OS in NSCLC patients with *SMARCA4* WT (N = 1327) vs. *SMARCA4*-mutated patients (N = 163): *SMARCA4* WT 25.0 months vs. *SMARCA4*-mutated 15.6 months (HR: 0.064; *p* < 0.001) [21]. However, in the immunotherapy-treated cohort of *SMARCA4*-mutated (N = 57) and WT patients (N = 475), which included single-agent checkpoint therapy or ICI in combination with a Cytotoxic T-Lymphocyte Antigen 4 (CTLA-4) inhibitor, there were no significant differences in *SMARCA4* WT vs. *SMARCA4*-mutated patients: PFS (3.1 vs. 2.1 months, HR: 0.93; *p* = 0.62) or OS (12.4 months vs. 11 months, HR: 0.83; *p* = 0.25) [21]. When getting more granular and looking at different types of *SMARCA4* mutations class 1 (N = 26) vs. class 2 (N = 31), there was no statistical OS difference in outcomes after receiving PDL-1 therapy when comparing *SMARCA4*-mutated class 1 [nonsense, frameshift, and splice site] (6.7 months; *p* = 0.11) vs. WT patients (N = 275, 12.4 months) vs. *SMARCA4*-mutated class 2 missense mutations (11.9 months, HR: 1.03; *p* = 0.87) [21]. 

When examining class 1 or class 2 mutations regardless of the type of treatment, Fernando et al. compared patients with *SMARCA4* WT (N = 2194) vs. *SMARCA4* NSCLC mutations in four categories: (a) homozygous truncated (N = 102), (b) homozygous nontruncated (N = 101), (c) heterozygous truncated (N = 16), and (d) heterozygous nontruncated (N = 49] [11]. There was a significant OS difference when comparing *SMARCA4*-mutated homozygous truncated vs. WT (7.9 vs. 16.3 months, HR: 1.85; *p* < 0.0001) but no significant differences when comparing other *SMARCA4*-mutated categories vs. *SMARCA4* WT [11].

Fernando et al. conducted a separate analysis when looking at the same four *SMARCA4* mutation categories but for patients who received immunotherapy (nivolumab, pembrolizumab, atezolizumab, or durvalumab) at any time during their cancer treatment [11]. This ICI treatment analysis compared patients with *SMARCA4* WT (N = 1069) vs. *SMARCA4* NSCLC mutations in four categories: (a) homozygous truncated (N = 38), (b) homozygous nontruncated (N = 41), (c) heterozygous truncated (N = 5), and (d) heterozygous nontruncated (N = 23). Similar to the non-treatment-specific OS analysis, there was a significant OS difference when comparing *SMARCA4*-mutated homozygous truncated vs. *SMARCA4*-WT (9.9 vs. 19.5 months, HR: 1.62; *p* = 0.01), but no significant differences when comparing WT with the other *SMARCA4*-mutated categories in the ICI-treated patients [11].

In a different study with the largest number of *SMARCA4* mutations in this review (N = 3305), the authors focused on clinicopathologic characteristics of patients with *SMARCA4* mutations and BRG1-deficient NSCLC [6]. Even though this study had a large number of *SMARCA4* patients, they only performed a *SMARCA4* mutation PFS analysis (N = 16) with 11 patients on chemotherapy and five on CIT. These patients had extremely short PFS of only 38 and 35 days, respectively [6]. 

Several studies included OS multivariate analysis across stages to address the outcomes of patients with *SMARCA4* mutations in the context of Stage III vs. Stage IV disease. One study conducted by Liang et al. revealed that individuals with *SMARCA4* mutations experienced significantly poorer survival in both locally advanced (Stage III) (N = 21) and metastatic (Stage IV) (N = 69) scenarios when compared to those with WT status [5]. The median survival for *SMARCA4*-mutated vs. WT patients was notably lower in Stage III (23.73 vs. 29.43 months, *p* < 0.01) and Stage IV (11.93 vs. 28.23 months, *p* < 0.01) [5]. To support this finding in Stage III patients, Talvitie et al.’s multivariate analysis on *SMARCA4*-mutated patients (N = 31) also revealed worse OS vs. WT patients (HR:1.93; *p* = 0.04) [23]. Regardless of stage, both authors conducted a multivariate analysis showing that *SMARCA4* WT (N = 221) patients had a better OS than mutated patients [(N = 105), 28.23 months vs. 12.17 months; *p* < 0.001] and *SMARCA4*-mutated patients had a worse OS than WT (HR: 3.522; *p* < 0.002) [5,23].

In association with *SMARCA4* mutations and clinico-genomic biomarkers across two different studies, tumor mutational burden (TMB) and brain metastasis in Anaplastic Lymphoma Kinase (ALK)-positive NSCLC were evaluated [24,25]. In exploring the relevance of TMB, Xu et al. conducted an NSCLC adenocarcinoma multivariate analysis [24]. The four arms were as follows: (a) *SMARCA4* WT, TMB Low (N = 761), (b) *SMARCA4* WT, TMB High (N = 722), (c) *SMARCA4*-mutated, TMB Low (N = 26), and (d) *SMARCA4*-mutated, TMB High (N = 69) [24]. The results showed a statistically better overall survival for *SMARCA4* WT, TMB High (*p* = 0.00019) [24]. In the context of brain metastasis, one study examined clinico-genomic outcomes in patients with (ALK)-positive NSCLC treated with alectinib [25]. They determined that *SMARCA4*-mutated patients (N = 3) with brain metastasis were the only group with co-mutations (*SMARCA4*/*ALK*) to do statistically worse across a multivariate analysis (HR: 8.76; *p* = 0.009).

When addressing *SMARCA4* mutation OS differences in a multivariate analysis of biological sex, two OS results were mixed between the sexes [5,26]. Pan et al. conducted a retrospective multivariate analysis, which included a cohort across 21 medical centers. There were 44 NSCLC patients with *SMARCA4* mutations, and men (N = 25) had a significantly worse OS than women (N = 19) (2.75 months vs. inestimable, HR:14.2; *p* = 0.02) [26]. The authors shared that one reason for the inestimable survival may be the low sample size of *SMARCA4* NSCLC patients. In the second study, the authors conducted a multivariate analysis, which resulted in statistically worse OS for women (N = 88) vs. men (N = 25) (HR: 4.1; *p* = 0.04) [5].

There were two NSCLC analyses of the OAK study involving atezolizumab. In the first OAK study analysis, Wang et al. discovered that *SMARCA4* was more likely to be seen in the negative PDL-1 expression group, but there was no statistical difference in OS when comparing atezolizumab vs. docetaxel in *SMARCA4*-mutated (N = 100) whether the patients were PDL-1 high or negative (HR = 0.67; *p* = 0.256) [27]. In addition, there was no difference in OS in *SMARCA4*-mutated NSCLC patients (N = 39) vs. *SMARCA4* WT (N = 491) whether the patients were PDL-1 high or negative when treated with atezolizumab (HR = 1.185, *p* = 0.513) [27]. In a second OAK uni-variate analysis, *SMARCA4*-mutated patients (N = 18) receiving atezolizumab did not fare worse than *SMARCA4* WT patients (N = 181) (HR: 1.70; *p* = 0.064) [28].

In the analysis by Velut et al., NSCLC patients with *SMARCA4* mutations (N = 7) did worse than WT (N = 70), independent of whether they received ICI therapy or not (HR: 3.2; *p* = 0.006) [29]. Interestingly, OS rates at 1 year were not significantly different between *SMARCA4*-mutated and WT patients but dropped significantly at 2 (58% vs. 74%) and 5 years (37% vs. 53%). In addition, patients were significantly younger upon diagnosis with *SMARCA4* mutation vs. without (61.9 years vs. 66.6 years; *p* = 0.01) [29]. A second single-institution analysis demonstrated a numerical, but not statistical, PFS benefit (6.3 vs. 3.9 months, HR: 0.64; *p* = 0.15) in *SMARCA4* WT (N = 130) vs. *SMARCA4*-mutated (N = 16) patients receiving ICIs (87% received combination ICI-chemotherapy) [30]. 

Three additional studies demonstrated significantly worse OS outcomes for NSCLC patients with *SMARCA4* mutations than wild-type patients across a multivariate analysis. In the first study, Yang et al.’s analysis resulted in worse OS for mutated (N = 5) vs. WT (N = 32) patients (4.5 months vs. 13.3 months, HR: 2.86; *p* = 0.031) [31]. The second multivariate analysis obtained a worse PFS (3 vs. 8 months; *p* = 0.007) for *SMARCA4*-mutated (N = 4) vs. WT patients (N = 99), and the results concluded that *SMARCA4* mutations were independently associated with a worse OS (HR: 3.098; *p* = 0.038) [32]. The last *SMARCA4* mutation analysis highlighted that the differences in all-cause mortality remained significant between the adenocarcinoma NSCLC patients that were mutated (N = 21) vs. wild-type (N = 204) (HR: 2.06; *p* = 0.003) [33]. 

### 3.3. SMARCA4/KRAS Co-Mutations

Seven NSCLC publications contained *SMARCA4*/*KRAS* co-mutation survival outcomes [8,21,22,33,34,35,36]. Two of these studies had specific *SMARCA4*/*KRAS* point mutation outcomes (i.e., *KRAS* G12C and G12D), and one analyzed survival with co-mutations in patients across clinical characteristics (i.e., brain and liver mets) [34,35,36]. Moreover, two different co-mutated analyses were performed by Alessi et al., highlighting robust results across many types and combinations of ICIs and chemotherapy [21,22]. Across various treatments, Schoenfeld et al.’s multivariate *SMARCA4*/*KRAS* co-mutation analysis resulted in worse statistical OS regardless of class 1 (N = 58) or 2 alterations (N = 52) (class 1: HR: 1.59 and class 2: HR: 2.75 vs. WT; *p* < 0.001) [8].

Negrao et al. produced the only publication focusing on NSCLC patients with co-mutations and *KRAS* G12C inhibitors (sotorasib or adagrasib). All NSCLC patients in their study had *KRAS* G12C mutations [35]. *SMARCA4*/*KRAS* G12C co-mutated patients (N = 18) had significantly worse PFS than *SMARCA4* WT/*KRAS* G12C-mutated patients (N = 213) (1.6 vs. 5.4 months, HR: 3.04; *p* < 0.001) and had a significantly worse OS (4.9 months vs. 11.8 months, HR: 3.07; *p* < 0.001) [35]. In relation to these survival outcomes, *SMARCA4* was one of the three tumor suppressor genes significantly enriched in the early progressing subgroup (*p* = 0.001) [35].

The second study that contained granular co-mutation outcomes (*KRAS* G12D) demonstrated statistically worse PFS for NSCLC patients; PFS: *SMARCA4*/*KRAS* G12D-co-mutated (N = 8) vs. *SMARCA4* WT/*KRAS* G12D-mutated (N = 49) (1.5 vs. 4.0 months; *p* = 0.0039) [34]. However, OS was only numerically worse but not statistically significant: *SMARCA4*/*KRAS* G12D-co-mutated vs. *SMARCA4* WT/*KRAS* G12D-mutated (6.1 vs. 17.3 months; *p* = 0.4202) [34]. 

Boiarsky et al. demonstrated that more than two times the number of patients had co-mutated *SMARCA4*/*KRAS* NSCLC Stage IV disease upon diagnosis (14%, N = 214) vs. Stage I (5%, N = 83) (*p* = 0.038), and these co-mutated patients had worse outcomes across patterns of metastatic spread (i.e., the brain) [36]. For example, OS was significantly worse for *SMARCA4*/*KRAS*-co-mutated (N = 33) patients with brain metastasis vs. *SMARCA4* WT/*KRAS*-mutated (N = 231) patients (7.4 months vs. 15.0 months, HR: 2.1; *p* = 0.0003) [36]. This HR was similar to *SMARCA4*/*KRAS*-co-mutated (N = 25) patients with liver metastasis vs. *SMARCA4* WT/*KRAS*-mutated (N = 155) patients (5.2 months vs. 13.2 months, HR: 2.1; *p* = 0.00015) [36].

*SMARCA4*-mutated patients had worse CIT treatment outcomes than *SMARCA4* WT patients in both *KRAS* WT and *KRAS*-mutated non-squamous NSCLC [22]. In reference to how co-mutated *SMARCA4*/*KRAS* non-squamous patients (N = 44) fared vs. *SMARCA4* WT/*KRAS*-mutated patients (N = 232), they fared even worse: PFS: *SMARCA4*/*KRAS*-mutated vs. *SMARCA4* WT/*KRAS*-mutated (2.2 vs. 6.2 months, HR: 2.39; *p* < 0.001), OS: *SMARCA4*/*KRAS*-mutated vs. *SMARCA4* WT/*KRAS*-mutated (6.6 vs. 14.6 months, HR: 2.52; *p* < 0.001) [22]. In the second Alessi et al. analysis with single-agent ICI or ICI-combination with CTLA-4, *KRAS*-mutant NSCLC was the most common co-mutation in the *SMARCA4* mutant subset (N = 17) [21]. The *SMARCA4*/*KRAS*-co-mutated patients had significantly shorter PFS (1.4 versus 4.1 months, HR: 0.25; *p* < 0.001) and OS (3 versus 15.1 months, HR: 0.29; *p* < 0.001) compared with NSCLC patients with *SMARCA4* WT/*KRAS* mutations (N = 159). Thus, the presence of a *SMARCA4*/*KRAS* co-mutation may confer a worse outcome to ICIs [21].

The last analysis from Liu et al. demonstrated significantly worse OS for all four *SMARCA4*/*KRAS*-mutated adenocarcinoma NSCLC cohorts across ICI-treated and non-ICI-treated NSCLC patients [37]. In Cohort A, *SMARCA4*/*KRAS*-co-mutated vs. *SMARCA4* WT/*KRAS*-mutated patients had worse OS (15.73 vs. 19.73 months) in a non-immunotherapy cohort in The Cancer Genome Atlas (TCGA) (HR: 2.32, *p* = 0.047). In Cohort B, *SMARCA4*/*KRAS*-co-mutated vs. *SMARCA4* WT/*KRAS*-mutated patients had worse OS (5.2 vs. 6.5 months) in a non-immunotherapy MSK-CT cohort (HR: 1.95, *p* = 0.015). In Cohort C, *SMARCA4*/*KRAS* co-mutated vs. *SMARCA4* WT/*KRAS*-mutated patients (1.73 vs. 4.22 months) had worse PFS in an immunotherapy MSK-IO cohort (HR: 2.15; *p* = 0.048). In Cohort D, *SMARCA4*/*KRAS*-co-mutated vs. *SMARCA4* WT/*KRAS*-mutated patients had worse OS in the immunotherapy Wake Forest cohort (HR: 11.98, *p* = 0.0018) [37].

## 4. Discussion

This *SMARCA4* NSCLC critical review focuses on survival outcomes across clinicopathological characteristics, *KRAS* co-mutations, and treatment with ICIs within twenty-one peer-reviewed articles. Eleven of thirteen (85%) *SMARCA4* mutation survival analyses in this review demonstrated significantly worse overall survival for *SMARCA4*-mutated NSCLC patients regardless of cancer treatment type. There were somewhat mixed results across *SMARCA4*-mutated treatment analysis for patients receiving ICIs and ICIs in combination with chemotherapy or CTLA-4 treatment. Three of the seven ICI analyses had worse OS for *SMARCA4*-mutated patients, three had no OS differences, and one outlier study highlighted that the patients with *SMARCA4* mutations did better on ICIs regardless of the class of *SMARCA4* mutation. Regarding *SMARCA4*/*KRAS* co-mutations, all seven treatment analyses (four with ICIs, two with non-ICIs, and one with *KRAS* G12C inhibitors) indicated that co-mutated *SMARCA4*/*KRAS* NSCLC patients consistently had significantly worse OS than *SMARCA4* WT/*KRAS*-mutated cohorts [8,21,22,34,35,36,37].

### 4.1. SMARCA4 Molecular Sub-Types and Genomic Features

In the largest *SMARCA4*-mutated survival analysis in this review (N = 407), the authors determined that class 1 truncating mutations were the strongest independent factor for significantly worse OS for NSCLC patients [8]. Class 1 mutations are typically associated with BRG1 protein loss compared to class 2 mutations, not losing any BRG1 protein expression (81% vs. 0%, *p* < 0.001) [8]. This lack of protein expression was also seen in an analysis completed at Massachusetts General Hospital (MGH), where 84% of patients with class 1 truncating mutations lacked BRG1 protein expression [6]. However, class 2 mutations (missense, nontruncating) also had worse OS, indicating that protein function could be impacted even though protein expression was evident [8].

As the only outlier in this review, *SMARCA4*-mutated NSCLC patients treated with ICI therapy had a longer OS (*p* = 0.01) [8]. In contrast, this ICI treatment OS benefit is not seen as appreciable when looking at *SMARCA4* WT vs. class 1 or 2 *SMARCA4* mutations. Alessi et al. point out that Schoenfeld’s results may differ from their extensive *SMARCA4* ICI therapy analysis due to imbalances in unreported baseline clinicopathological characteristics in the Schoenfeld publication, which could have altered the OS [21].

As further support, both Alessi et al. studies highlight *SMARCA4* mutations as an independent factor in a significantly worse outcome for NSCLC patients [21,22]. However, different conclusions arose in their two separate publication analyses of ICI treatment: (1) based on whether it was used in the first line with chemotherapy (CIT), or (2) in pre-treated patients as a single agent (ICI) or ICI/CTLA4 combination therapy. In the first-line setting, *SMARCA4* mutations were associated with significantly worse OS to first-line CIT treatment in non-squamous NSCLC (*SMARCA4*-mutated 8.1 months vs. *SMARCA4* WT 15.0 months (*p* < 0.001) [22]. In the pre-treatment setting, the authors did not find an association between *SMARCA4* mutational status and ICI treatment on PFS or OS, or whether the patient had a class 1 or 2 *SMARCA4* mutation [21].

When looking more granularly at truncated vs. nontruncated *SMARCA4* mutations or homozygous vs. heterozygous, there was a significantly worse OS when comparing *SMARCA4* homozygous truncated vs. WT (7.9 vs. 16.3 months, HR: 1.85; *p* < 0.0001) but no significant differences when comparing other *SMARCA4*-mutated categories vs. WT [11]. This significant OS difference was also demonstrated when looking specifically at ICI treatment when comparing homozygous truncated vs. WT (9.9 vs. 19.5 months, HR: 1.62; *p* = 0.01), but no significant differences were obtained when comparing WT with the other *SMARCA4*-mutated categories in the ICI-treated patients [11].

TMB is a genomic feature being analyzed in NSCLC across various ICI studies [38]. It is being reviewed as a possible biomarker for the use of ICIs in *SMARCA4*-mutated NSCLC [21]. Xu et al. showed a worse OS for NSCLC patients that are *SMARCA4*-mutated (TMB Low or TMB High) as compared to *SMARCA4* WT/TMB High (*p* = 0.00019) [24].

### 4.2. Clinicopathological Features

Multiple studies, including Liang et al. and Talvitie et al., conducted OS multivariate analyses across different stages, revealing consistently poorer survival for patients with *SMARCA4* mutations [5,23]. Liang et al.’s study demonstrated significantly worse OS in both locally advanced (Stage III) and metastatic (Stage IV) *SMARCA4*-mutated scenarios compared to WT status (Stage III (23.73 vs. 29.43 months, *p* < 0.01) and Stage IV (12.17 vs. 28.23 months, *p* < 0.01) [5]. Talvitie et al.’s analysis in Stage III patients further supported worse OS for *SMARCA4*-mutated patients [23]. Regardless of which stage, multivariate analyses consistently showed worse OS for *SMARCA4*-mutated patients, highlighting the impact of these mutations on survival outcomes.

This *SMARCA4* review illustrated statistically significant OS differences based on brain metastasis and mixed results based on biological sex [5,25,26]. In the context of brain metastases, Miao et al. investigated clinicogenomic outcomes in *ALK*-positive NSCLC patients treated with alectinib, finding that *SMARCA4*-mutated patients with brain metastases were the only group with co-mutations to exhibit statistically worse outcomes in a multivariate analysis (HR: 8.76; *p* = 0.009) [25]. In two multivariate analyses of the impact of biological sex on *SMARCA4* mutation OS differences, one analysis demonstrated significantly worse survival for men, and the other demonstrated worse survival for women [5,26]. Pan et al. reported that male NSCLC patients with *SMARCA4* mutations had a markedly worse OS than females (HR: 12.64; *p* = 0.002), while Liang et al.’s study highlighted a worse OS for women across a cohort of Stage IV *SMARCA4*-mutated patients (HR:14.2; *p* = 0.02) [5,26]. Although women had a statistically significant worse OS in the multivariate analysis, the authors share that one of the reasons for the possible large female survival discrepancy was due to lower numbers of females than males in the study (88 vs. 133) [5].

In the context of *SMARCA4* NSCLC patients treated with immune checkpoint inhibitors (ICIs), one study found that those with *SMARCA4* mutations had worse outcomes than WT patients regardless of ICI treatment or not (HR = 3.2; *p* = 0.006), and two separate OAK trial NSCLC analyses showed no OS differences with atezolizumab (ICI) vs. docetaxel in pre-treated NSCLC *SMARCA4*-mutated patients [27,28,29]. Three additional studies demonstrated significantly worse OS outcomes for NSCLC patients with *SMARCA4* mutations vs. WT patients across a multivariate analysis and various standards of care treatments [30,31,32].

### 4.3. SMARCA4/KRAS Co-Mutations

All seven *SMARCA4*/*KRAS* co-mutation NSCLC publications in this review showed either a significantly worse PFS or OS as compared to *SMARCA4* WT/*KRAS* mutation analyses across standard-of-care cancer treatments or within a specific treatment analysis (i.e., *KRAS* G12C inhibitor, ICIs, or non-ICIs) [8,21,22,34,35,36,37]. Two of these studies presented granular survival outcomes specific to two *KRAS* isoforms (G12C and G12D) [34,35]. Finally, one analysis demonstrated significantly worse survival for *SMARCA4*/*KRAS*-co-mutated patients as they presented at a higher rate of Stage IV NSCLC with a significantly higher propensity of brain or liver metastasis [36]. These higher rates of metastatic spread led to significantly worse survival for co-mutated *SMARCA4*/*KRAS* vs. *SMARCA4* WT/*KRAS*-mutated patients who either had brain or liver metastasis (i.e., brain metastasis 7.4 months vs. 15.0 months, HR: 2.1; *p* = 0.0003) [36].

*SMARCA4*/*KRAS* co-mutations are among the most common co-mutations within this review. Among the largest co-mutation analyses within the review, *SMARCA4*/*KRAS* was co-mutated at a rate of 36% in NSCLC [8]. Within this multi-variate analysis and across various treatments, the *SMARCA4*/*KRAS* co-mutation analysis by Schoenfeld et al. resulted in worse OS regardless of class 1 or 2 alteration [class 1: HR: 1.59, and class 2: HR: 2.75] vs. WT (*p* < 0.001) [8]. Cooper et al.’s PFS co-mutation *SMARCA4*/*KRAS* G12D analysis demonstrated significantly worse outcomes for co-mutated patients vs. *SMARCA4* WT/*KRAS* G12D-mutated patients (1.5 vs. 4.0 months; *p* = 0.0039) [34]. However, as the outlier in this critical review focusing on *KRAS* G12D, the *SMARCA4*/*KRAS* G12C co-mutation OS analysis did not result in a significant difference as compared to *SMARCA4* WT/*KRAS*-mutated NSCLC patients (6.1 vs. 17.3 months; *p* = 0.42) [34].

### 4.4. SMARCA4/KRAS Co-Mutation Treatment Analysis

Four studies reported six *SMARCA4*/*KRAS* co-mutation OS survival treatment analyses [21,22,35,37]. All six treatment analyses across various treatment types (i.e., ICI, non-ICI, or *KRAS* G12C inhibitor) resulted in a significantly worse OS (*p* < 0.05) for *SMARCA4*/*KRAS*-co-mutated NSCLC patients. Negrao et al. had the only publication focusing on *KRAS* G12C NSCLC patients with co-mutations and *KRAS* G12C inhibitors (sotorasib or adagrasib) [35]. NSCLC patients with *SMARCA4*/*KRAS* G12C co-mutations vs. *SMARCA4* WT/*KRAS* G12C mutations had significantly worse OS (4.9 months vs. 11.8 months, HR: 3.07; *p* < 0.001). In relation to these survival outcomes, *SMARCA4* was one of the three tumor suppressor genes significantly enriched in the early progressing subgroup (*p* = 0.001) [35].

Across two publications by Alessi et al., whether the co-mutated NSCLC patients were treated in the first line with CIT or in ≥ 2nd line with single-agent ICI in combination with CTLA-4, the patients had statistically worse OS [21,22]. Co-mutated *SMARCA4*/*KRAS* non-squamous CIT-treated patients had worse OS compared to *SMARCA4* WT/*KRAS*-mutated OS (6.6 vs. 14.6; *p* < 0.001) [22]. In the second Alessi et al. analysis with single-agent ICIs or an ICI combination with CTLA-4, co-mutated NSCLC patients had significantly worse OS compared with NSCLC patients with *SMARCA4* WT/*KRAS* mutations (3 months versus 15.1 months, HR: 0.29; *p* < 0.001) [21]. Thus, the authors concluded that *SMARCA4*/*KRAS* co-mutation may confer worse NSCLC survival outcomes to ICIs [21].

Liu et al.’s ICIs and non-ICIs treatment adenocarcinoma NSCLC analysis was associated with significantly worse OS or PFS across all four *SMARCA4*/*KRAS* co-mutated vs. *SMARCA4* WT/*KRAS*-mutated cohorts [37]. Cohort A and B *SMARCA4KRAS*-mutated patients were associated with worse OS when treated with non-ICIs (*p* = 0.047 and *p* = 0.015). In Cohort C and Cohort D, *SMARCA4KRAS*-mutated patients treated with immunotherapy had worse PFS and OS (Cohort C (PFS): *p* = 0.048 and Cohort D (OS): *p* = 0.0018) [37].

There are potential reasons why current treatments are not highly effective in treating *SMARCA4*/*KRAS* NSCLC co-mutations, as the tumor microenvironment (TME) plays an important role in metastatic NSCLC [39]. Regarding ICI therapy, Liu et al. used a signature gene panel to evaluate the level of immune cell type across each NSCLC individual [37]. They found that *SMARCA4*/*KRAS*-co-mutated patients had a significantly lower estimated proportion of Cluster of Differentiation 8 (CD8+) T-cells (*p* = 0.015) and activated CD4+ memory T cells (*p* = 0.0035) than *SMARCA4* WT/*KRAS*-mutated subjects [37]. This suggests that CD8+ T-cell function may be impacted by regulatory T cells (Tregs), which can lead to a more immunosuppressive tumor microenvironment landscape [39]. Beyond ICI monotherapy and looking at CIT, *SMARCA4* mutations can activate Nuclear Factor Erythroid 2-Related Factor 2 (NRF2) signaling, leading to the up-regulation of cytoprotective genes and enabling platinum therapy resistance [40].

## 5. Future Direction

The majority of *SMARCA4*-mutated NSCLC patient analyses within this review demonstrated a worse overall survival as compared to *SMARCA4* wild-type patients. Mutations in this gene should be explored as a prospective biomarker across ICI, CIT, and *KRAS* G12C inhibitor clinical trials. As each *SMARCA4*/*KRAS* co-mutated treatment analysis in this review showed a statistically worse OS or PFS than *SMARCA4* WT/*KRAS*-mutated patients, a prospective comprehensive immune cell analysis could be considered for *SMARCA4*-mutated and co-mutated *SMARCA4*/*KRAS* patients to understand better how these mutations and co-mutations impact the tumor microenvironment. Utilization of this prospective biomarker approach and immune cell data analysis could inform the design of future NSCLC clinical trials.

## 6. Limitations

The vast majority of mutation and treatment analyses (i.e., single agent and combination approaches) in this review are retrospective and vary in *SMARCA4* mutation sample size. The scope of the publications was limited to those in the English language published from 2018 through 2023. *KRAS* was the primary co-mutated gene included, rather than analyzing other possible co-mutations with *SMARCA4* (e.g., *KEAP1*/*STK11*). Given these limitations, this review’s strength is that it is one of the largest *SMARCA4* overall survival NSCLC reviews completed to date.

## 7. Conclusions

In one of the largest reviews to date of *SMARCA4*-mutated overall survival data in NSCLC, two consistent trends emerged from the twenty-one publications in scope: First, *SMARCA4* NSCLC mutated patients had statistically worse OS than *SMARCA4* WT patients. Secondly, NSCLC patients with *SMARCA4*/*KRAS* co-mutations tended to have significantly worse overall survival than *SMARCA4* WT/*KRAS*-mutated patients, regardless of treatment type, within each treatment analysis. Moreover, designing prospective clinical *SMARCA4*-mutated or *SMARCA4*/*KRAS*-co-mutated NSCLC trials to evaluate targeted therapies and immunotherapy may lead to a better understanding of how to improve cancer patients’ outcomes and survival rates. These findings may have implications for future research and clinical practice.

## Figures and Tables

**Table 1:** Summary of articles included in this critical review. (A) *SMARCA4* Mutations. (B) *SMARCA4*/*KRAS* Co-mutations.

**(A)**					
**Author**	**Publication Year**	**Treatment**	**NSCLC Mutation Analyzed**	**Number of NSCLC Patients with Mutation Analyzed**	**Outcomes/Relevance**
Schoenfeld	2020	Various SOC	*SMARCA4*	N = 292 mut [class 1 mut (N = 149) and class 2 (N = 143)]	In a multivariate analysis, both *SMARCA4* mut class 1 and 2 had worse OS (*p* < 0.001) vs. WT. Class 1 mut had worse OS compared to class 2 mut or WT (*p* < 0.001).
		ICI cohort	*SMARCA4*	N = 87 mut	OS improvement for *SMARCA4* mut with ICI treatment vs. without ICI treatment regardless of class 1 or class 2 mut status (*p* = 0.01).
					The unadjusted ICI analysis showed no difference in PFS (*p* = 0.74) or OS (*p* = 0.35) with class 1 mut vs. class 2 mut.
Alessi	2023	CIT	*SMARCA4*	114 mut	In multivariate analysis: worse OS for *SMARCA4* mut vs. WT (8.1 versus 15.0 months, HR: 1.70; *p* < 0.001) and worse PFS: (2.7 versus 6.1 months, HR: 1.62; *p* < 0.001).
Alessi	2021	Various SOC	*SMARCA4*	163 mut	*SMARCA4* mut had worse OS (15.6 months) vs. *SMARCA4* WT (25 months) (HR: 0.64; *p* < 0.001).
		ICI or ICI/CTLA4 cohort	*SMARCA4*	57 mut class 1 non-sense, frameshift, and splice site mut (N = 26) and class 2 missense mut (N = 31)	There were no significant differences in *SMARCA4* WT vs. mut: PFS (3.1 vs. 2.1 months; HR = 0.93, *p* = 0.62) or OS (12.4 vs. 11 months; HR = 0.83, *p* = 0.25 months).
					There was no OS difference in outcomes after receiving ICI therapy when comparing class 1 nonsense, frameshift, and splice site mut (6.7 months; *p* = 0.11) vs. WT patients (12.4 months) vs. class 2 missense mut (11.9 months, HR = 1.03, *p* = 0.87).
Fernando	2020	Various SOC	*SMARCA4*	*SMARCA4* mut in four categories: (1) homozygous truncated (N = 102), (2) homozygous nontruncated (N = 101), (3) heterozygous truncated (N = 16), (4) heterozygous nontruncated (N = 49)	There was a significant OS difference when comparing *SMARCA4* homozygous truncated mut vs. *SMARCA4* WT (7.9 vs. 16.3 months, HR: 1.85; *p* < 0.0001), but no significant differences when comparing the other *SMARCA4* mut categories with WT.
		ICI cohort		*SMARCA4* mut four categories: (1) homozygous truncated (N = 38), (2) homozygous nontruncated (N = 41), (3) heterozygous truncated (N = 5), (4) heterozygous nontruncated (N = 23)	There was a significant OS difference when comparing *SMARCA4* mut homozygous truncated vs. *SMARCA4* WT (9.9 vs. 19.5 months, HR: 1.62; *p* = 0.01), but no significant differences when comparing the other *SMARCA4* mut categories with WT.
Dagogo-Jack	2020	Chemo or CIT	*SMARCA4*	16 mut	Extremely short PFS for chemo and CIT treatment (38 and 35 days), respectively.
Liang	2023	Various SOC	*SMARCA4*	Stage III mut (N = 21), Stage IV mut (N = 69)	Median OS survival for *SMARCA4* mut vs. *SMARCA4* WT patients was notably lower in Stage III (23.73 vs. 29.43 months, *p =* <0.01) and Stage IV (11.93 vs. 28.23 months, *p =* <0.01).
			*SMARCA4*	221 mut women (N = 88), men (N = 25)	Multivariate analysis resulted in statistically worse OS for women vs. men (HR = 4.1, *p* = 0.04).
Talvitie	2022	Various SOC	*SMARCA4*	135 mut	*SMARCA4* mut patients had a worse OS than *SMARCA4* WT (HR: 3.522; *p* < 0.002).
			*SMARCA4*	Stage III mut (N = 31)	Stage III multivariate analysis *SMARCA4* mut patients had a worse OS vs. WT patients (HR:1.93; *p* = 0.04).
Xu	2023	Various SOC	*SMARCA4*	Four Arms: (1) *SMARCA4* WT, TMB Low (N = 761), (2) *SMARCA4* WT, TMB High (N = 722), (3) *SMARCA4* mut, TMB Low (N = 26), and (4) *SMARCA4* mut, TMB High (N = 69).	Statistically better OS for *SMARCA4* WT/TMB High (*p* = 0.00019).
Miao	2023	Alectinib (ALK)	*SMARCA4*/*ALK*	3 mut	In a multivariate analysis, *SMARCA4* mut patients with brain metastasis were the only co-mut with *ALK* with worse outcomes (HR: 8.76; *p* = 0.009).
Pan	2023	Various SOC	*SMARCA4*	44 mut: women (N = 19), men (N = 25)	Multivariate analysis with *SMARCA4* mut: men had a significantly worse OS than women (2.75 months vs. un-estimable, HR:14.2; *p* = 0.02).
Wang	2021	Atezo (ICI) or docetaxel	*SMARCA4*	100 mut	OAK analysis: there is no statistical difference in OS when comparing atezolizumab vs. docetaxel in *SMARCA4* mut, whether PDL-1 high or negative (HR = 0.67; *p* = 0.256).
			*SMARCA4*	39 mut	No difference in OS with PDL-1 high vs. low when treated with atezo (HR = 1.185, *p* = 0.513)
Marinelli	2020	Atezo (ICI)	*SMARCA4*	18 mut	OAK uni-variate analysis: *SMARCA4*-mutated patients receiving atezo did not fare worse than *SMARCA4* WT patients (HR: 1.70; *p* = 0.064).
Velut	2022	Nivolumab or pembro	*SMARCA4*	7 mut	SD-NSCLC patients had a shorter OS than patients with non-SD-NSCLC, considering either from the date of diagnosis (*p* = 0.01, HR: 2.9) or from the date of immunotherapy (*p* = 0.006, HR: 3.2). OS rates at 1 year were not significantly different between *SMARCA4* mut and WT patients but dropped significantly at 2 (58% vs. 74%) and 5 years (37% vs. 53%).
Chang	2022	ICI (87% of patients)	*SMARCA4*	16 mut	No significant PFS difference in *SMARCA4* WT vs. mut (6.3 vs. 3.9 months, HR: 0.64; *p* = 0.15).
Yang	2023	Various SOC	*SMARCA4*	5 mut	*SMARCA4* mut patients had a worse OS compared to WT patients (4.5 months vs. 13.3 months; HR: 2.86; *p* = 0.031). Significantly worse PFS for *SMARCA4* mut vs. WT (2.3 months vs. 8.1 months, HR: 2.80; *p* = 0.035).
Wang	2021	SOC	*SMARCA4*	4 mut	In multivariate analysis, *SMARCA4* mut patients fared worse than *SMARCA4* WT (HR = 3.098, *p* = 0.038) and had worse PFS (3 vs. 8 months, *p* = 0.007).
Le Fleur	2019	Various SOC	*SMARCA4*	21 mut	In a multivariate all-cause mortality analysis, *SMARCA4* mut were independently associated with a worse OS prognosis (HR: 2.06, *p* = 0.003).
**(B)**					
**Author**	**Publication Year**	**Treatment**	**NSCLC Mutations Analyzed**	**Number of NSCLC Patients with Mutations Analyzed**	**Outcomes/Relevance**
Cooper	2022	Various SOC	*SMARCA4*/*KRAS* G12D co-mut	8 co-mut	Significantly worse PFS for co-mut patients; PFS: *SMARCA4*/*KRAS* G12D mut vs. *SMARCA4* WT/*KRAS* G12D mut (1.5 vs. 4.0 months; *p* = 0.0039).
			*SMARCA4 WT*/*KRAS* G12D mut	49 mut	No significant difference in OS: *SMARCA4*/*KRAS* G12D co-mut vs. *SMARCA4* WT/*KRAS* G12D mut (6.1 vs. 17.3 months; *p* = 0.4202).
Negrao	2023	Sotorasib or adagrasib	*SMARCA4*/*KRAS* G12C co-mut	20 co-mut	*SMARCA4*/*KRAS* co-mut patients had significantly worse PFS than *SMARCA4* WT/*KRAS* mut (1.6 months vs. 5.4, HR: 3.04; *p* < 0.001) and had a significantly worse OS (4.9 months vs. 11.8 months, HR: 3.07; *p* < 0.001).
			*SMARCA4 WT*/*KRAS* G12C	214 mut	
Boiarsky	2023	Various SOC	*SMARCA4*/*KRAS* co-mut	33 co-mut	OS was significantly worse for *SMARCA4*/*KRAS* co-mut patients with brain metastasis vs. *SMARCA4* WT/*KRAS*-mutated (7.4 months vs. 15.0 months, HR: 2.1; *p* = 0.0003).
			*SMARCA4* WT/*KRAS* mut	231 mut	Liver metastasis: *SMARCA4*/*KRAS* co-mut had worse outcomes vs. *SMARCA4* WT/*KRAS*-mutated (5.2 months vs. 13.2 months, HR: 2.1; *p* = 0.00015).
Alessi	2021	CIT	*SMARCA4*/*KRAS* co-mut	44 co-mut	Significantly worse OS (6.6 vs. 14.6 months; HR: 2.52, *p* < 0.001) and PFS (2.2 vs. 6.2 months; HR: 2.39, *p* < 0.001) in *SMARCA4*/*KRAS* co-mut vs. *SMARCA4* WT/*KRAS* mut patients.
			*SMARCA4* WT/*KRAS* mut	232 mut	
Alessi	2023	Single-agent ICI or ICI combined with CTLA-4	*SMARCA4*/*KRAS* co-mut	17 co-mut	*SMARCA4*/*KRAS* co-mut had significantly shorter PFS (1.4 vs. 4.1 months, HR: 0.25; *p* < 0.001) and OS (3 vs. 15.1 months, HR: 0.29; *p* < 0.001) compared with *SMARCA4* WT/*KRAS* mut patients.
			*SMARCA4 WT*/*KRAS* mut	159 mut	The presence of a *SMARCA4*/*KRAS* co-mut may confer a worse outcome to ICIs.
Schoenfeld	2020	Various SOC	*SMARCA4*/*KRAS* co-mut	110 co-mut 58 co-mut (class 1 *SMARCA4*) 52 co-mut (class 2 *SMARCA4*)	Multivariate *SMARCA4*/*KRAS* co-mut analysis resulted in worse statistical OS regardless of class 1 or 2 alterations. [Class 1: HR: 1.59, and class 2: HR: 2.75] vs. WT (*p* < 0.001).
Liu	2021	Various SOC	*SMARCA4*/*KRAS* co-mut	Cohort A: *SMARCA4*/*KRAS* co-mut (N = 9) vs. *SMARCA4* WT/*KRAS* mut (N = 146)	Cohort A: *SMARCA4*/*KRAS* co-mut vs. *SMARCA4* WT/*KRAS* mut had worse OS (15.73 vs. 19.73 months) in non-immunotherapy cohort in The Cancer Genome Atlas (TCGA) (HR: 2.32, *p* = 0.047).
			*SMARCA4 WT*/*KRAS* mut	Cohort B: *SMARCA4*/*KRAS* co-mut (N = 34) vs. *SMARCA4* WT/*KRAS* mut (N = 280)	Cohort B: *SMARCA4*/*KRAS* co-mut vs. *SMARCA4* WT/*KRAS* mut had worse OS (5.2 vs. 6.5 months) in non-immunotherapy MSK-CT cohort (HR: 1.95, *p* = 0.015).
				Cohort C: *SMARCA4*/*KRAS* co-mut (N = 9) vs. *SMARCA4* WT/*KRAS* mut (N = 68)	Cohort C: *SMARCA4*/*KRAS* co-mut vs. *SMARCA4* WT/*KRAS* mut (1.73 vs. 4.22 months) had worse PFS in immunotherapy MSK-IO cohort (HR: 2.15; *p* = 0.048).
				Cohort D: *SMARCA4*/*KRAS* co-mut (N = 2) vs. *SMARCA4* WT/*KRAS* mut (N = 16)	Cohort D: *SMARCA4*/*KRAS* co-mut vs. *SMARCA4* WT/*KRAS* mut had worse OS in immunotherapy Wake Forest cohort (HR: 11.98, *p* = 0.0018).

Atezo, atezolizumab; chemo, chemotherapy; co-mut, co-mutation; CTLA-4, Cytotoxic T-Lymphocyte Antigen 4; ICI, Immune Checkpoint Inhibitor; HR, Hazard Ratio; mets, metastasis; mut, mutation; OS, Overall Survival; PD1, Programmed Cell Death protein 1; PDL-1, Programmed Cell Death Ligand-1; pembro, pembrolizumab; PFS, Progression Free Survival; SOC, Standard of Care; WT, Wild-type.

## Data Availability

No new data were created or analyzed in this study. Data sharing is not applicable to this article.
